# Prevalence of Autism Spectrum Disorders in Ecuador: A Pilot Study in Quito

**DOI:** 10.1007/s10803-015-2559-6

**Published:** 2015-08-30

**Authors:** Laura M. S. Dekkers, Norbert A. Groot, Elena N. Díaz Mosquera, Ivonne P. Andrade Zúñiga, Martine F. Delfos

**Affiliations:** PICES (PICOWO), Psychological Institute for Consultation, Education, and Scientific Research, Joseph Haydnlaan 2A, 3533 EA Utrecht, The Netherlands; Department of Developmental Psychology, University of Amsterdam, Weesperplein 4, 1018 XA Amsterdam, The Netherlands; Centro Meta, P.O. Box 17-21-622, Quito, Ecuador; Facultad de Psicología, PUCE, Pontificia Universidad Católica del Ecuador, Av. 12 de octubre 1076 y Roca, Quito, Ecuador; Universidad Central del Ecuador, Ciudadela Universitaria, Av. América, Quito, Ecuador

**Keywords:** Autism, ASD, Prevalence, Epidemiology, Ecuador, South America

## Abstract

This research presents the results of the first phase of the study on the prevalence of pupils with Autism Spectrum Disorder (ASD) in regular education in Quito, Ecuador. One-hundred-and-sixty-one regular schools in Quito were selected with a total of 51,453 pupils. Prevalence of ASD was assessed by an interview with the rector of the school or its delegate. Results show an extremely low prevalence of 0.11 % of pupils with any ASD diagnosis; another 0.21 % were suspected to have ASD, but were without a diagnosis. This low prevalence suggests that children and adolescents with ASD are not included in regular education in Quito. These results are discussed in the light of low diagnostic identification of ASD and low inclusion tolerance.

## Introduction

Pervasive Developmental Disorders–in accordance with the DSM-5 (APA 2013) further to be referred to as Autism Spectrum Disorders (ASD)—are one of the most studied developmental disorders, but are still not well understood. ASD is characterized by impairments in functioning in the social environment and slow processing of information. The newest research insights on its etiology have shown that ASD is highly heritable (Eapen 2011) and indicate a delay in brain development (Bastiaanse et al. 2011; Hazlett et al. 2006; Hedvall et al. 2014; Hua et al. 2011; Roberts et al. 2013; Whitehouse et al. 2011). Survey studies propose the best estimate for the prevalence of ASD as being 70–90/10,000 (Fombonne 2009), with a male to female ratio of 4:1 (Duchan and Patel 2012). However, in studies after 2004, this ratio has increased to 100–250/10,000 (Isaksen et al. 2013). Most research on (the prevalence of) ASD has taken place in North America, Europe, and Japan. One of the problems in studying the true prevalence of ASD, however, is that the classification of ASD, like all psychiatric diagnoses, is based on behavior, and different circumstances can lead to autistic *behavior* without being ASD emanating from a *specific genetic pattern* (Delfos 2006, 2010). People in general expect ASD to be based on a specific genetic pattern, a ‘nature’ problem. Often this is not the case and behaviors associated with ASD—e.g., social skills deficits, anxiety or sensory sensitivities may also be triggered by environmental exposures, like neglect (Rutter et al. 1999) or a traumatic experience. In fact, for at least 70 % of the cases the underlying genetic cause is unknown (Schaaf and Zoghbi 2011). In addition, there is phenotypic overlap with other neurodevelopmental disorders such as Attention Deficit Hyperactivity Disorder (ADHD; Craig et al. 2015; Kiser et al. 2015) and Obsessive Compulsive Disorder (OCD; Jacob, et al. 2009), which may add to diagnostic uncertainty, particularly in regions that are lacking specialists trained in ASD diagnosis. This is why Stessman et al. (2014) plead for developing a ‘genotype-first’ approach to define (genetic) subtypes in complex diseases as ASD.

Autism was first described, independently from each other, by Kanner (1943) and Asperger (1938, 1944/1991). It is supposed to be of all times and all cultures, but in most non-western countries no research with respect to autism/ASD has been performed yet. Ecuador is one of those countries. In South America only three studies on the prevalence of ASD are known (Lejarraga et al. 2008; Montiel-Nava and Peña 2008; Paula et al. 2011). They show a very low prevalence and are not all population based (cf. Paula et al. 2011). Firstly, in a Venezuelan clinical-based study a prevalence of 17/10,000 was found among 3–9 year old children (Montiel-Nava and Peña 2008). Secondly, in an Argentinian study among 839 children under the age of 5, recruited at three different health care centers, an ASD prevalence of about 1.3 % was found (Lejarraga et al. 2008). Finally in a study conducted at a typical town in Southeast of Brazil, the ASD prevalence was found to be 27/10,000 (Paula et al. 2011).

Groot from Centro Meta (Ecuador) and Delfos from the Psychological Institute for Consultation, Education, and Scientific Research (PICES, The Netherlands) performed one study in two phases on ASD in Ecuador (Groot and Delfos 2008). It was a pilot study on early detection of ASD based on the expertise of parents about their child with ASD. The results showed that Ecuadorian parents know at a very early stage that there is something the matter, but it takes them many years before they get the diagnosis of ASD for their child.

In 2011, Delfos started to establish a Universities Autism Expertise Group (UAEG; see Delfos 2010) adapted to Ecuador (Delfos and Groot 2011). The same year, in collaboration with Pontificia Universidad Católica del Ecuador (PUCE), PICOWO and Centro Meta, the project *‘*Educational reality of children and youngsters with Autism Spectrum Disorder (ASD), in educational institutions of Quito’ (PAE–Prevalence of Autism in Ecuador—project) was initiated. The first phase of the PAE project was to examine the prevalence of ASD in children and adolescents in Ecuador.

The situation in Ecuador with respect to psychiatric diagnoses is underdeveloped and first a study on the overall view about ASD in Ecuador was performed (Delfos and Groot 2011). The most important city of Ecuador is Quito, the capital of Ecuador, and with its over 1.6 million estimated number of inhabitants (INEC 2010) it is the second largest city with respect to population. During this initial study (Delfos and Groot 2011) and clinical work with children and adolescents with ASD, we observed that in Quito almost no specialized teams are available to perform a diagnostic assessment of ASD. Most diagnoses come from the very few child pediatricians or child neurologists. Also, there is very little day care or special services for children with ASD available. The association of parents of children with ASD is in development. Finally, insights from research on the etiology of ASD only begin to reach the people and as Ecuador became a Catholic country because of the Hispanic conquerors, ASD was for a long time, and in many areas still is, considered to be a punishment of God.

The aim of the current study was to get an estimate of ASD diagnoses in children and adolescents aged between of five and fifteen at regular schools in Quito. To be able to establish the prevalence of ASD in Quito it was clear that this first research could only be considered as a start and that the real prevalence would probably be hidden because of the lack of services that can assess ASD. To think that it would be possible to establish the prevalence of ASD in Ecuador seemed too ambitious, but we had to find a way to prove this. However, in the period during which this research was carried out disabilities came into focus through Lenin Moreno, vice-president of Ecuador from 2008 till 2012, who was nominated for the Peace Nobel Prize in 2012, because of his work for disabled people. ASD was not yet paid attention to in 2010 and little was known about autism, but it started to become a governmental focus from 2011 on.

## Methods

### Site

Ecuador is a country with a large diversity in many aspects. Most native inhabitants originate from a mixture of Hispanic and Indian cultures. The main language is Spanish, next to many other languages. Ecuador has more than 14 million inhabitants (INEC 2010), of which over 1.6 million are estimated to live in the capital city of Quito (INEC 2010). Quito is unique in the world in the sense that concerning sea level, it is the second highest capital built at 9350 feet/2800 m. This has physical consequences for the population in the sense of a slightly higher blood pressure.

The Ecuadorian law states that all children should attend school (Salamanca Agreements 1994) and the country is beginning to develop *inclusive education*. All children are supposed to attend regular schools, and schools are not allowed to refuse children because of their handicap. Law and practice do not overlap yet, for instance in the rural areas and in poor areas in the cities, parents need their children to help them to earn money in order to survive. These parents are now granted money by the government for sending their children to school. Still, many children, often girls and very often children with disabilities, are not attending school. The endeavor of the government is to make school education possible for all children.

Because of the law requiring inclusive education, and granting parents money to make their children attend regular schools, it was decided for the first phase of the prevalence research PAE project to identify the cases of ASD in regular schools. The city of Quito was chosen, because of its large population and this region was considered as having the best-organized administration of the four regions, within the 24 provinces of Ecuador. As the region of Quito is considered to be well-organized at the administration level, this would mean that the pupils with ASD in regular schools in Quito would be expected to be registered in the school administration.

### Study Design

The design was based on regular schools in the metropolitan district Quito. Regular schools are assumed to be attended by all children from 5 to 15 years old for half a day. This would make it possible to show the supposed prevalence of ASD, at least in regular schools.

The regular schools were identified by using the databanks from the Ministry of Education. The metropolitan district Quito has 1259 regular schools. Basic criteria for a representative sample of schools were: regular schools from the metropolitan district of Quito, Spanish spoken, morning education (as school education in Quito is normally half a day and not fulltime). Sample size was based on a power analysis, where N as the total sample size of 1259 schools; Z_a_^2^ was the 90 % confidence interval of 1.64; υ was the proportion based on previous research, set to 0.5 μ was the complementary proportion, also set to 0.5; and ɛ is the permitted error rate, set to 0.06. This analysis yielded a sample size n = 162 schools, which were randomly dawn, stratified on type of school (i.e., public, religion based, municipal, and particular), from all 1259 regular schools. The number of included schools and associated total number of pupils with respect to their type is summarized in Table 1. As can be verified, stratification on type of school resulted in sampling more Private followed by Public schools compared with Religion based and Municipal schools, χ2(3) = 177.01, *p* < .001. Also, most pupils came from Private followed by Public schools compared with Religion based and Municipal schools, K(3) = 34.28, *p* < .001.

**Table 1:** Characteristics of included schools and associated total number of pupils

	Percentage of included schools	Number of included schools (*n*)	Chi square test	Total number of pupils (*N*)	Kruskal–Wallis test
Type of school			*χ* ^*2*^(3) = 177.012 *p* < .001		*K*(3) = 34.282 *p* < .001
Public	28.57	46		27,603	
Religion based	3.73	6		2667	
Municipal	1.24	2		119	
Private	66.45	107		21,064	
Location in Quito			*χ* ^*2*^(4) = 10.894 *p* = .028		*K*(4) = 2669 *p* = .609
North	18.01	29		6267	
Centre-North	29.81	48		17,318	
Centre	14.29	23		7172	
Centre-South	19.25	31		11,517	
South	18.63	30		9179	
Size of the school			*χ* ^*2*^(2) = 9.329 *p* = .009		*K*(2) = 139.051 *p* = .009
Small	44.10	71		22,409	
Medium	31.06	50		20,073	
Large	24.84	40		8971	
Pedagogical focus			*χ* ^*2*^(5) = 152.752 *p* < .001		*K*(5) = 19.985 *p* < .001
Constructionist	50.31	81		29,584	
Modern	22.36	36		9102	
Governmental	8.07	13		3005	
Traditional	2.48	4		497	
Otherwise	6.83	11		3046	
Not specified	9.94	16		6219	
Total	100.00 %	161		51,453	

Chi square and Kruskal–Wallis tests were used to test whether schools and number of pupils within each school, respectively, were equally distributed over the different levels of the different characteristics of the schools (i.e., type of school, geographical part in Quito the school is located, size of the school, pedagogical focus of the school). Size of the school was defined by, small: <101 pupils, medium: 101–400 pupils, large: >400 pupils

As one school no longer existed at the start of the research—which happens quite frequently in Ecuador, schools come and go—the final sample consisted of *n* = 161 schools, that all agreed to participate. From these schools, the following demographical information was gathered after inclusion in the study and accounted for in the statistical analyses: the geographical part of Quito the school is located (Quito being a long ‘ribbon’, constructed on the flanks of two mountain chains, the regions were: North, Centre-North, Centre, Centre-South, South; size of the school (i.e., small: <101 pupils, medium: 101–400 pupils, large: >401 pupils), and pedagogical focus of the school (i.e., constructionist; modern; official governmental guidelines; traditional). As mentioned before, this demographic information was gathered after inclusion of the school in the study; the sampling procedure was not stratified on these demographics. However, for transparity, the number of included schools and associated total number of pupils with respect to the geographical part of Quito the school is located, its size, and its pedagogical focus are summarized in Table 1. As can be verified, the number of included schools was dependent on the geographical part in Quito the school is located, *χ*^*2*^(4) = 10.89, *p* = .028. However, this was not true for the total number of included pupils, *K*(4) = 2,67, *p* = .609, which was equally distributed across all five geographical parts. In addition, more small followed by medium compared to large sized schools were included, *χ*^*2*^(2) = 9.33, *p* = .009. Also, most pupils came from small followed by medium compared to large sized schools, *K*(2) = 139.05, *p* = .009. Finally, the number of included schools was dependent on its pedagogical focus, *χ*^*2*^(5) = 152.75, *p* < .001; so was the total number of included pupils, *K*(5) = 19.96, *p* < .001. That is, schools with a constructionist focus were overrepresented, encompassing most pupils. However, it should be noted that although this analysis indicates a dominance of the constructionist focus, this may not be taken to suggest a truly biased pedagogical focus, as the constructionist focus, reported by the schools, is basically prescribed by the government, but not always followed by schools.

The rectors, or their delegates, of all schools were interviewed with a semi-structured questionnaire, covering demographics of the school and fourteen questions concerning prevalence and suspicion of children with ASD, knowledge about ASD, age and gender of the child with/suspected of ASD, and refusal of school attendance of children with/suspected of ASD. As the accessibility of diagnostic services that can assess ASD is extremely low in Ecuador, we could not use methods for finding cases that are used in developed countries. As far as we discovered, there are only two neuro-pediatricians that play a role in assessing developmental disorders in Quito and one institute where assessment of these disorders is possible. We met the people involved in diagnostics in Quito, to find out as much as we could about the diagnosis of ASD in Ecuador. Based upon this, we developed a design through which (1) the information could be trusted most and (2) children diagnosed with ASD could be found. The result was the administration of a semi-structured questionnaire at regular schools in Quito. With respect to the first criteria (i.e., information that could be trusted the most), we opted for the region Quito as in Quito the governmental organization and administration is considered the best of Ecuador. Furthermore, we opted for regular schools. The administration of institutions is up to the level of governmental standards. The schools follow strict governmental rules for administration. So, the administration of schools is supposed to be the best organized in Ecuador. That is, the administration of schools is quite precise, and visiting schools we discovered that each time we had to show our passport and credentials to be received at a school. Implementing the Salamanca agreements, the government is very keen on inclusive education and expects the children with ADS to be included in regular schools. Together, this would mean that the information obtained at regular schools in Quito could be trusted most. With respect to the second criteria (i.e., children with ASD could be found), our choice for the region Quito seemed warranted as in Quito the accessibility of diagnostic services is high as compared to the rest of the country.

### The Case Identification

The cases of ASD were identified by the school administration. As cases of ASD were accepted pupils who had been given an official DSM-III (APA 1980) or DSM-IV (APA 1994) diagnosis of (Classical) Autism, Pervasive Development Disorder-Not Otherwise Specified (PDD-NOS), Disintegrative Disorder of Childhood, Asperger Disorder (syndrome)/(High Functioning Autism -HFA), Rett disorder, by a medical authority (mostly diagnosis by a child-neurologist) in the field of ASD.

## Results

Response on school interviews was 100 % for the 161 schools, with a total of 51,453 pupils from 5 to 15 years old. Most schools (150 out of 161 = 93 %) had some knowledge about ASD. Significantly more schools were familiar with the concept of ASD compared to not familiar, *Z* = 10.88, *p* < .001. Whether schools knew about ASD did not depend on either their location in Quito (North, Centre-North, Centre, Centre-South, South), *χ*^*2*^(4) = 1.63, *p* = .821, nor their size (small, medium, large), *χ*^*2*^(2) = 2.26, *p* = .342.^1^

Of all schools, 33 schools (20.3 %) reported that they had at least one pupil with an official diagnosis of ASD. There were significantly less schools with one pupil with an ASD diagnosis as compared to schools without at least one pupil with an ASD diagnosis, *Z* = 7.41, *p* < .001. Whether or not schools reported that they had at least one pupil with an ASD diagnosis was not dependent on which part of Quito the school was located nor on its pedagogical focus (constructionist; modern; official governmental guidelines; traditional), *χ*^*2*^(4) = 7.38, *p* = 0116 and *χ*^*2*^(4) = 4.77, *p* = .443, respectively. It was, however, dependent on size of the school, *χ*^*2*^(3) = 6.92, *p* = .033, with the probability of reporting at least one pupil with an ASD diagnosis decreasing with the size of the school.

Of the total number of pupils within all schools (*N* = 51,453) only 57 pupils had an official diagnosis of ASD. This brings the prevalence of ASD in Quito, as reported by schools for regular education, to 11.07 out of 10,000, which is 0.11 %. The distribution of the reported diagnoses of these 57 pupils was: Classical autism: 13; Asperger syndrome: 13; PDD-NOS: 30; Disintegrative Disorder of Childhood: 1; Rett Disorder: 0. Details with respect to the reported distribution of these pupils across the schools with different characteristics are outlined in Table 2.

**Table 2:** Reported prevalence of pupils with a diagnosis or suspected of an Autism Spectrum Disorders (ASD) in regular schools in Quito, Ecuador

	Classical Autism	Asperger syndrome	PDD-NOS	Disintegrative disorder of childhood	Rett disorder	Total ASD diagnosis	Total suspected of ASD
*Type of school*							
Public	1	0	2	0	0	3	32
Religion based	0	0	0	0	0	0	7
Municipal	0	0	0	0	0	0	0
Private	12	13	30	1	0	56	126
*Location in Quito*							
North	0	4	4	0	0	8	35
Centre-North	4	5	12	1	0	22	35
Centre	0	0	1	0	0	1	9
Centre-South	4	2	10	0	0	16	15
South	5	2	3	0	0	10	14
*Size of the school*							
Small	10	7	23	1	0	41	30
Medium	2	5	3	0	0	10	34
Large	1	1	4	0	0	6	44
*Pedagogical focus*							
Constructionist	4	6	18	0	0	28	62
Modern	4	4	2	1	0	11	24
Governmental	3	2	1	0	0	6	7
Traditional	0	0	8	0	0	8	1
Otherwise	0	1	0	0	0	1	4
Not specified	2	0	0	0	0	2	10
Total	13	30	13	0	1	57	108

Size of the school was defined by, small: <101 pupils, medium: 101–400 pupils, large: >400 pupils

In accordance with previous research (Werling and Geschwind 2013), and as shown in Table 3, ASD was reported significantly more frequently among boys (*N* = 47) as compared to girls (*N* = 10), *Z* = 4.77, *p* < .001; ratio boys/girls: 4.7 times more boys than girls. Mean age of boys (*M* = 7.98, *SD* = 4.24) and girls (*M* = 8.66, *SD* = 3.38) with an ASD diagnosis was not significantly different, *t*(55) = −0,48, *p* = .635.

**Table 3:** Gender differences of pupils with a diagnosis or suspected of an Autism Spectrum Disorder (ASD)

	Number of pupils	*M* Age (*SD*)
Diagnosed with ASD	57	8.10 (4.08)
Boys	47	7.98 (4.24)
Girls	10	8.66 (3.38)
Suspected of ASD	108	9.13 (4.13)
Boys	83	8.67 (3.89)
Girls	25	10.64 (4.58)

*M* = Mean; *SD* = Standard deviation

Out of the 161 schools, 59 schools (36.6 %) mentioned that they thought that at their school there was at least one pupil who did not have an ASD diagnosis, but should have been diagnosed with ASD. Together, these were 108 pupils in total. Details with respect to the reported distribution of these pupils across the schools with different characteristics are outlined in Table 2. In addition, as can again be verified in Table 3, when ASD was suspected, this was more often in boys (*N* = 83) as compared to girls (*N* = 25), Z = 5.49, *p* < .001, and boys (*M* = 8.67; *SD* = 3.89) suspected to have ASD were younger as compared to girls (*M* = 10.64; *SD* = 4.58), *t*(105) = −2,04, *p* = .049. Whether or not schools thought that at their school at least one pupil was present that did not have a diagnosis of ASD, but met criteria for such a disorder, was not dependent on either their location in Quito, *χ*^*2*^(4) = 6.26, *p* = .180, size of the school, *χ*^*2*^(2) = 2.34, *p* = .337, or its pedagogical focus, *χ*^*2*^(2) = 7.26, *p* = .203.

Out of all schools (*N* = 161), 14 schools (8.7 %) reported that they had at least once refused a boy or girl with ASD to their school, because of his or her behavior.

## Discussion

This study aimed to investigate the prevalence of ASD in regular schools in Quito, which was found to be 0.11 %, that is 11/10,000 persons. This is much lower than the expected 70–90/10,000, that was based on a summary of epidemiological studies on the prevalence of ASD (Fombonne 2009). It was also lower than the prevalence found in a Brazilian study, being 27/10,000 (Paula et al. 2011). Even when we take the cases with a diagnosis of ASD and suspected to have ASD together, the prevalence is still very low, 0.33 %. We suspect that this prevalence does not reflect the true prevalence of children and adolescents with ASD in Ecuador and think that it is rather an *incidence* indicator as a result of access to diagnostics facilities, that is, the probability of occurrence of a given medical condition within a specified period of time. We propose that the found percentage of 0.11 % reflects the *current probability* of receiving a diagnosis of ASD in Quito, Ecuador, rather than the proportion of the population that has ASD.

There are several reasons to suspect that the found prevalence is not reflecting the true prevalence of ASD in Ecuador. The most important are:

1. Quito is not representative for Ecuador, but the prevalence would be highest in Quito, compared to the rest of Ecuador, as accessibility of diagnostic services is highest in Quito and prevalence estimates are often higher in urban versus rural areas (Williams et al. 2006), resulting in an over- instead of underestimation of the prevalence of ASD in Ecuador.
2. Accessibility of diagnostic services that can assess ASD is very low in Ecuador, certainly for the rural areas.
3. Knowledge about ASD is not widely spread, nor refined or up to date in the country. School representatives therefore may not have been able to recognize cases of ASD.
4. Universities are not familiar with ASD; in general there is no knowledge or research about ASD.
5. There are very few professionals trained in assessing ASD.
6. Autism is often not recognized as such and considered a punishment of God, leaving the family in shame hiding their child with problem behavior.

The low prevalence is probably most influenced by the low diagnostic identification of ASD in Ecuador. Problems with this identification are that the diagnosis is mostly on the initiative of parents (Delfos and Groot 2011), and that there is a low accessibility of diagnostic services in Quito, however Quito is still much better than other parts of Ecuador. There is a bias against severe cases in the regular schools, for instance the tendency of exclusion of pupils with the comorbidity ASD and mental retardation and/or speech problems.

This study involved only the regular schools in Quito. Because accessibility of diagnostic services is much lower in other parts of the country, the prevalence could be expected to be much lower in other parts of Ecuador without representing thereby a realistic prevalence. In addition, to have a better view on prevalence of ASD in Quito, the special schools of Quito should be studied (see, Ouellette-Kuntz et al. 2007; Maenner and Durkin 2010). The ASD prevalence in special education was the second part of phase 1 of the PAE project, including 11 special schools and centers, and a total of 1195 pupils; publication of the results is in preparation. We can expect a higher prevalence at these schools, because there is a trend for higher and increasing prevalence of autism in special education in areas with low prevalence of autism (Maenner and Durkin 2010). Finally, epidemiological studies outside the school are needed to assess the prevalence of children and adolescents with (suspected) ASD that are not be able to attend (special) education.

We trained the psychology students to heighten the reliability of the answers of the questionnaire. The part of the questionnaire asking for the facts about pupils with a diagnosis of ASD seem reliable, as the school is obliged to register these facts. We suspect that the part where the rectors, or their delegates (often the school pedagogue) were asked whether they suspected to have children in their school who would meet the criteria for an ASD diagnosis, however, is less reliable. We do not know much about the specific knowledge or training about ASD of the persons interviewed, but we do know that knowledge and training is scarcely available in Ecuador. The question about refusing to admit children because of their behavior is probably even less reliable, because the schools are obliged to admit all children, and will not easily provide information indicating the contrary.

As the idea of inclusion in regular schools is only just reaching public awareness, there still is low inclusion tolerance. This is reflected in both the extremely low found prevalence of 0.11 % and in what is thought to be a false response outcome of a low refusal—of pupils with ASD in regular schools—only 8.7 % of the schools admitted to have at least once refused a pupil with ASD. For governmental politics the extremely low prevalence of children and adolescents with ASD at regular schools is of interest, because it seems to show that children with ASD are not included in regular education.

The low identification of ASD represents a heavy burden for children with ASD and their families. Developing classification and diagnoses is of utmost importance. Therefore trained and experienced professionals are necessary. In developed countries the accessibility of diagnostic services resulted in long waiting lists, which had to be worked through by less experienced and less trained professionals, possibly resulting in a false ASD diagnosis. This also happened because the DSM-IV (APA 1994) is not specific enough (for a review see, Volkmar et al. 2012) and certainly PDD-NOS could have lead to false diagnoses. The problem of false diagnoses was addressed by Rutter et al. (1999) with the Romanian orphans, suffering from neglect instead of autism. Especially in developing countries this is a possibility to take into account. This problem should be met in helping countries develop ASD diagnosis on a trustworthy basis. Moreover, some concerns should be expressed about a possible increase of diagnoses without further access to treatment services or school.

The higher incidence of pupils suspected of having ASD, but no official diagnosis, shows that in the schools there is some awareness of ASD. The schools could therefore be one of the places for developing autism help in Ecuador. The ration male to female is somewhat higher (4.7:1) but close to the general ratio of 4:1 (Duchan and Patel 2012; also see, Werling and Geschwind 2013), and could reflect the true ratio.

To conclude, the first phase of the PAE project shows an extremely low prevalence of 11/10,000 of pupils with ASD in regular schools in Quito, Ecuador. The children with ASD could be attending special education, but it is more probable that although the prevalence will be higher in special education, it is not conform to the expected prevalence coming from developed countries. We cannot think of a reason the condition of ASD itself would have a lower prevalence in Ecuador. We propose it is the low awareness of ASD and very limited accessibility of autism assessment that fosters the low prevalence. This means that the situation of children, adolescents, and adults with ASD in Ecuador is probably very difficult, similar to the situation before Asperger (1938, Asperger 1944/1991) and Kanner (1943) discovered the condition.

## Footnotes

1 For all analyses, it was tested whether results were dependent on the geographical part of Quito the school is located (i.e., North, Centre-North, Centre, Centre-South, South), size of the school (i.e., small: <101 pupils, medium: 101–400 pupils, large: >400 pupils), pedagogical focus of the school (i.e., constructionist; modern; official governmental guidelines; traditional), and type of school (i.e., public, religion based, municipal, private). If for either of these variables no results are reported, this is due to the fact that in some instances analyses could not be carried out, as the results of zero observations.

## References

[CR1] American Psychological Association, APA (1980). *Diagnostic and statistical manual of mental disorders* (3rd Ed.) Washington, DC: American Psychological Association.

[CR2] American Psychological Association, APA (1994). *Diagnostic and statistical manual of mental disorders* (4th Ed.) Washington, DC: American Psychological Association.

[CR3] American Psychological Association, APA (2013). *Diagnostic and statistical manual of mental disorders* (5th Ed.) Washington, DC: American Psychological Association.

[CR4] Asperger H (1938). Das psychisch abnorme Kind. Wiener Klinischen Wochenzeitschrift.

[CR6] Asperger, H. (1944/1991). ‘Autistic psychopathy’ in childhood. In U. Frith (Ed.), *Autism and Asperger syndrome* (pp. 37–92). Cambridge: Cambridge University Press. (Original work published in 1944).

[CR7] Bastiaanse JA, Thioux M, Nanetti L, van der Gaag C, Ketelaars C, Minderaa R, Keysers C (2011). Age-related increase in inferior frontal gyrus activity and social functioning in autism spectrum disorder. Biological Psychiatry.

[CR8] Craig F, Lamanna AL, Margari F, Matera E, Simone M, Margari L (2015). Overlap between Autism Spectrum Disorders and Attention Deficit Hyperactivity Disorder: Searching for distinctive/common clinical features. Autism Research.

[CR9] Delfos MF (2006). A strange world – Autism, Asperger’s Syndrome and PDD-NOS. A guide for parents, partners, professional Carers, and people with ASDs..

[CR10] Delfos, M. F. (2010). *Fact finding mission on autismin Bosnia and Herzegovina*. Retrieved Oct 27, 2013 form http://www.mdelfos.nl.

[CR11] Delfos, M. F. & Groot, N. A. (2011). *Informe. Incentivar la asistencia a personas con autism en el Ecuador. Report 04.* (Report. Building autism help in Ecuador.) Retrieved Sept 21, 2014 from www.mdelfos.nl/ecuador.

[CR12] Duchan E, Patel DR (2012). Epidemiology of Autism Spectrum Disorders. Pediatric Clinics of North America.

[CR13] Eapen V (2011). Genetic basis of autism: Is there a way forward?. Current Opinion in Psychiatry.

[CR14] Fombonne E (2009). Epidemiology of pervasive developmental disorders. Pediatric Research.

[CR15] Groot, N. A., & Delfos, M. F. (2008). *Het versterken van de rol van de familie in de deskundigheid over hun autistische familielid*. (Strengthening the role of the family in their expertise on their autistic relative.) Retrieved Oct 27, 2013 from http://www.mdelfos.nl.

[CR16] Hazlett HD, Poe MD, Gerig G, Gimpel Smith R, Piven J (2006). Cortical gray and white brain tissue volume in adolescents and adults with autism. Biological Psychiatry.

[CR17] Hedvall A, Westerlund J, Fernell E, Holm A, Gillberg C, Billstedt E (2014). Autism and developmental profiles in preschoolers: Stability and change over time. Acta Paediatica.

[CR18] Hua X, Thompson PM, Leow AD, Madsen SK, Caplan R, Alger JR, Levitt JG (2011). Brain growth rate abnormalities visualized in adolescents with autism. Human Brain Mapping.

[CR19] Instituto Nacional de Estadistica y Censos, INEC (2010). Resultatos del censo 2010. Retrieved Jan 3, 2014, from http: www.ecuadorencifras.gob.ec/resultados/.

[CR20] Isaksen J, Diseth TH, Scholberg S, Skjeldal OH (2013). Autism Spectrum Disorders—Are they really epidemic?. European Journal of Pediatric Neurology.

[CR21] Jacob S, Landeros Weisenberger A, Leckman JF (2009). Autism spectrum and obsessive–compulsive disorders: OC behaviors, phenotypes and genetics. Autism Research.

[CR22] Kanner L (1943). Autistic disturbances of affective contact. Nervous Child.

[CR23] Kiser DP, Rivero O, Lesch KP (2015). Annual Research Review: The (epi) genetics of neurodevelopmental disorders in the era of whole genome sequencing–unveiling the dark matter. Journal of Child Psychology and Psychiatry.

[CR24] Lejarraga H, Menendez AM, Menzano E, Guerra L, Biancato S, Pianelli P, Contreras MM (2008). Screening for developmental problems at primary care level: A field programme in San Isidro. Argentina. Paediatric and Perinatal Epidemiology.

[CR25] Maenner MJ, Durkin MS (2010). Trends in prevalence of autism on the basis of special education data. Pediatrics.

[CR26] Montiel-Nava C, Peña JA (2008). Epidemiological findings of pervasive developmental disorders in a Venezuelan study. Autism.

[CR27] Ouellette-Kuntz H, Coo H, Lloyd JEV, Kasmara L, Holden JJA, Lewis MES (2007). Trends in special education code assignments for autism: Implications for prevalence estimates. Journal of Autism and Developmental Disorders.

[CR28] Paula CS, Ribeiro SH, Fombonne E, Mercadante MT (2011). Brief Report: Prevalence of Pervasive Developmental Disorder in Brazil: A Pilot Study. Journal of Autism Developmental Disorders.

[CR29] Roberts TP, Lanza MR, Dell J, Qasmieh S, Hines K, Blaskey L, Berman JI (2013). Maturational differences in thalamocortical white matter microstructure and auditory evoked response latencies in autism spectrum disorders. Brain Research.

[CR30] Rutter M, Andersen-Wood L, Beckett C, Bredenkamp D, Castle J, Groothues C, English and Romanian Adoptees (ERA) Study Team et al (1999). Quasi-autistic pattern following severe early global privation. Journal of Child Psychology and Psychiatry.

[CR31] Salamanca Agreements (1994). *The Salamanca statement and framework for action on special needs education.* Unesco. Retrieved Sept 21, 2014 from http://unesdoc.unesco.org/images/0009/000984/098427eo.pdf.

[CR32] Schaaf CP, Zoghbi HY (2011). Solving the autism puzzle a few pieces at a time. Neuron.

[CR33] Stessman HA, Bernier R, Eichler EE (2014). A genotype-first approach to defining the subtypes of a complex disease. Cell.

[CR34] Volkmar FR, Reichow B, McPartland J (2012). Classification of autism and related conditions: Progress, challenges, and opportunities. Dialogues in Clinical Neuroscience.

[CR35] Werling DM, Geschwind DH (2013). Sex differences in autism spectrum disorders. Current Opinion in Neurology.

[CR36] Whitehouse AJO, Maybery MT, Hickey M, Sloboda DM (2011). Brief report: Autistic like traits in childhood predict later age at menarche in girls. Journal of Autism and Developmental Disorders.

[CR37] Williams JG, Higgins JP, Brayne CE (2006). Systematic review of prevalence studies of autism spectrum disorders. Archives of Disease in Childhood.

